# Dosimetry, Toxicity and Stability Associated With a Hyaluronic Acid‐Based Prostate‐Rectal Spacing Device During Prostate Radiation Therapy: A Retrospective Comparative Analysis

**DOI:** 10.1002/jmrs.70125

**Published:** 2026-09-22

**Authors:** Fadila Najem, Bridget Smith, Sary Chen, Lucinda Wood, Asmaa El‐Heneidy, Lloyd Smyth, Hodo Haxhimolla, Pat Bowden

**Affiliations:** ^1^ Icon Cancer Centre Canberra Bruce Australian Capital Territory Australia; ^2^ Icon Cancer Centre Revesby Revesby New South Wales Australia; ^3^ Griffith Biostatistics Unit Griffith University Brisbane Queensland Australia; ^4^ School of Medicine and Dentistry Griffith University Brisbane Queensland Australia; ^5^ Icon Group South Brisbane Queensland Australia; ^6^ Department of Urology National Capital Private Hospital Canberra Australian Capital Territory Australia; ^7^ University of Canberra Bruce Australian Capital Territory Australia; ^8^ College of Health and Medicine Australian National University Canberra Australian Capital Territory Australia

**Keywords:** hyaluronic acid, prostate, radiation therapy, spacer, symmetry, toxicity

## Abstract

**Introduction:**

Hypofractionated radiation therapy (HFRT) for localised prostate cancer may increase gastrointestinal (GI) toxicity. This study evaluated spacer geometry, dosimetric and clinical outcomes associated with the use of a stabilised hyaluronic acid (sHA)‐based rectal spacer, when inserted prior to HFRT, compared to a non‐spacer cohort.

**Method:**

This was a retrospective comparative cohort study of patients receiving HFRT (60 Gy in 20 fractions) between July 2019 and December 2023 at two centres from a single institution. Prostate‐rectum spacing was measured at apex, mid‐gland, and base levels at simulation as baseline and on five cone beam CT scans during treatment. Rectal dose‐volume metrics and planning target volume (PTV) coverage were compared. Toxicities were retrospectively graded using CTCAE Version 5.0 for participants with a minimum follow‐up of 3 months.

**Results:**

Ninety‐one patients were included in this study; 64 with sHA‐based spacers and 27 without spacing. The mean baseline spacing was 0.8, 1.0 and 0.9 cm at apex, mid‐gland and base, respectively, and this separation remained stable throughout treatment. The spacer cohort demonstrated significantly lower rectal doses across all metrics except V60Gy and achieved slightly higher PTV coverage (D98%: 57.8 vs. 57.3 Gy, *p* < 0.001). Acute GI toxicity (grade ≥ 1) was lower in the spacer cohort (23.5% vs. 48.0%; OR = 0.33, *p* = 0.05), with grade ≥ 2 toxicity in 2.9% versus 20.0%. Genitourinary toxicity rates were comparable between cohorts. No complications associated with spacer insertion were recorded.

**Conclusions:**

sHA‐based rectal spacers provided physically stable spacing throughout treatment, facilitating significant reductions in rectal dose and a favourable toxicity profile. This study adds further evidence to support the use of sHA‐based spacers in prostate HFRT.

## Graphical abstract

Rectal spacers composed of stabilised hyaluronic acid facilitated a favourable toxicity profile compared to no spacing during prostate radiation therapy. Acute GI toxicity (grade ≥ 1) was lower in the spacer cohort (23.5% vs. 48.0%; odds ratio = 0.33, *p* = 0.05) with grade ≥ 2 toxicity substantially lower (2.9% vs. 20.0%).

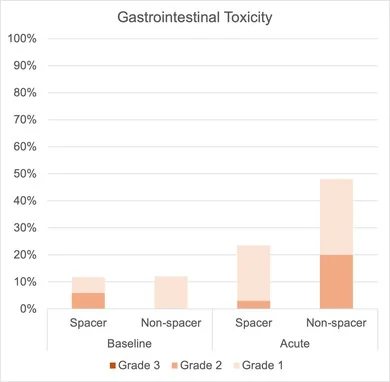

## 1 Introduction

The utilisation of hypofractionated radiation therapy (HFRT) in the setting of localised prostate cancer has increased substantially over the past decade in Australia [1, 2]. HFRT provides a shorter course of treatment (usually 4–5 weeks) compared to conventionally fractionated radiation therapy (usually 7–9 weeks), making it more convenient—especially for people who travel from regional areas to receive treatment. While HFRT is supported by level one evidence—being non‐inferior in terms of cancer control outcomes and survival [3, 4]—the risk of Grade 2 or higher acute gastrointestinal (GI) toxicity may be increased by up to approximately 10% [5].

Various types of rectal spacers, which increase the physical separation between the rectum and the prostate, have been shown to reduce the rate of both acute and late GI toxicity [6]. Stabilised hyaluronic acid (sHA)‐based spacers have emerged as a promising alternative to traditional hydrogel‐based spacers, offering potential advantages such as enhanced formability during insertion, clear visibility on ultrasound and reversibility if needed using hyaluronidase [7].

Although rectal spacing in prostate HFRT is well established, clinical data on the use of sHA‐based spacers remains relatively limited. A recent randomised trial, limited to 6 months follow up, reported significantly improved rectal dosimetry and significantly reduced acute (3 months post HFRT) grade ≥ 2 GI toxicities [8]. A small single arm Phase 2 study (*n* = 36) also reported a low rate of grade ≥ 2 GI toxicity (12%) within a 36‐month follow up period, with no grade ≥ 3 toxicities reported [9]. Other studies report on dosimetry and safety following spacer insertion with no post‐radiotherapy toxicity outcomes reported [10].

In this retrospective study, we present both dosimetric and clinical outcomes for a cohort of men with localised prostate cancer treated with HRFT following insertion of a sHA‐based rectal spacer. These data are compared to a cohort of men also receiving HFRT but without any form of rectal spacing. The aim of this study was to evaluate differences in dosimetry and toxicity outcomes between the cohorts, as well as to provide novel insight into the symmetry and stability of the sHA‐based spacer throughout the course of treatment. We hypothesised that the use of a sHA‐based spacer during prostate RT is associated with more favourable prostate‐rectum separation, which is (1) maintained over the course of treatment, (2) associated with an improved rectal dose distribution and (3) associated with a reduced rate of gastrointestinal toxicity related to RT.

## 2 Methods

### 2.1 Study Design, Setting, Participants

This is a retrospective comparative cohort study of men with localised prostate cancer receiving HFRT at two sites from the same institution between July 2019 and December 2023. This study was approved by the Bellberry Human Research Ethics Committee (approval number: 2023‐10‐1252) under a waiver of consent model.

Participants eligible for inclusion in this study were over 18 years old at the time of treatment, prescribed a course of prostate HFRT (60 Gy in 20 fractions), treated using volumetric modulated arc therapy (VMAT), and either had a sHA‐based rectal spacer inserted prior to treatment planning (spacer cohort) or had no form of rectal spacing (control cohort).

### 2.2 Spacer Injection and Treatment Planning

Participants in the spacer cohort had biodegradable sHA‐based gel (Barrigel, Santa Barbara, CA, USA) injected into the perirectal space using a standard procedure similar to that previously described [7]. In brief, the injection procedure was performed by a urologist under general anaesthesia with transrectal ultrasound guidance, in the dorsolithotomy position with legs in stirrups. Three fiducial markers were also implanted in the prostate (both in the spacer and non‐spacer cohorts). Prior to injection, the urologist reviewed the participant's MRI report and index lesion location to customise the spacer placement, ensuring optimal anatomical separation.

Following spacer injection, participants underwent MRI and CT scans approximately 5–7 days post‐injection to facilitate treatment planning. T2‐weighted MRI images were fused with CT datasets to enhance anatomical accuracy. All scans were performed according to departmental protocol, including maintenance of a full bladder and empty rectum to ensure consistency and reproducibility.

Treatment plans were generated using Eclipse v16.1 (Varian Medical Systems, Palo Alto, CA, USA). The radiation oncologist (RO) delineated the clinical target volumes (CTV), which included the prostate and proximal 1 cm of the seminal vesicles, based on the fused MRI and CT datasets according to standard local clinical guidelines. The planning target volume (PTV) was a 1 cm isotropic expansion of the prostate and seminal vesicles, except for posteriorly where the margin was 5 mm. The rectum, bladder, and sHA spacer were contoured by radiation therapists according to standard local clinical guidelines. Radiotherapy typically commenced 10–14 days following the spacer injection.

### 2.3 Data Collection and Outcome Measures

Disease history, treatment prescriptions, and clinical outcomes were extracted from each participant's electronic medical record (EMR). Toxicity data were collected from referral documents and clinician documentation in the EMR, with GI and genitourinary (GU) toxicities retrospectively graded using CTCAE Version 5.0, unless the CTCAE term and grade were specified at the time of assessment by the treating clinician. Only participants with a minimum of 3 months follow‐up data were included in the toxicity analysis.

Baseline PTV and rectal dose‐distribution data were retrieved from the treatment planning system; minimum dose to 98% of the PTV (D98%), and the percentage volume of the rectum receiving specific doses between 30 and 60 Gy (V30Gy, V40.8Gy, V50Gy, V52.8Gy, V54Gy, V57Gy, and V60Gy). Additionally, the rectal maximum dose was assessed both as a point dose and as D0.035cc. The rectum was contoured from the recto‐anal junction inferiorly to the rectosigmoid junction superiorly.

Separation between the rectum and the prostate was assessed at three levels; mid‐gland (central superior–inferior slice of the prostate), base (0.5 cm inferior to the most superior slice of the prostate), and the apex (0.5 cm superior to the most inferior slice of the prostate). Three lateral measurements per level were taken; midline as well as at 1.0 cm to the right and left of midline to assess spacer symmetry. Baseline spacing was assessed based on the fused MRI‐CT dataset used for treatment planning. Separation between the rectum and prostate during treatment was assessed from cone beam CT (CBCT) scans conducted prior to treatment. Five CBCTs were assessed per participant (fractions 1, 5, 10, 15, and 20).

The sHA spacer, rectum, and prostate were contoured on each CBCT dataset using the following method. CBCT datasets were fused to the treatment planning dataset with the sHA spacer, rectum, and prostate copied to the CBCT dataset and edited or recontoured based on daily anatomical variations. Edits and recontouring for the sHA spacer on the CBCT were performed primarily in the sagittal view using the breast window level to enhance soft tissue visibility. Rectal contours were either edited directly or recontoured every 2–3 slices using the interpolation function. The prostate generally required minimal editing due to consistent alignment achieved through fiducial matching, reducing the need for manual adjustments. This structured approach enabled reproducible and anatomically consistent measurement of rectum‐prostate separation across participants.

### 2.4 Statistical Analysis

Categorical demographic and toxicity data were described using proportions and percentages. Continuous variables were described using a mean and standard deviation, or median and first/third quartile values, as appropriate.

Independent‐samples *t*‐tests were used to assess the relationship between the use of sHA‐based spacer and rectal dose distribution. Mixed‐effects linear regression with participant entered as a random effect was used to investigate the association between the use of sHA‐based spacer during prostate RT and stable prostate‐rectum separation over the course of RT and to assess asymmetry between left and right sides at each timepoint and level. Robust standard errors were applied to account for the presence of heteroskedasticity. A joint Wald test of the timepoint coefficients and post‐estimation contrasts was used to formally assess whether the outcome varied across different timepoints. Post‐estimation contrasts were then used to examine pairwise differences between specific timepoints. Statistical significance was set at *p* < 0.05. Analyses were performed with Stata version 14.2 software (StataCorp, College Station, Texas).

## 3 Results

### 3.1 Demographics

A total of 91 patients from two sites were included in this retrospective analysis. Sixty‐four and 27 patients were included in the spacer and non‐spacer cohorts, respectively. Demographics and baseline clinical characteristics for both groups are summarised in Table 1. Most patients in both groups received treatment to the prostate and seminal vesicles (98.4% and 88.9% in the spacer and non‐spacer cohorts, respectively). The index lesion location was distributed similarly between both cohorts.

**TABLE 1:** Participant characteristics by group.

Variable	Spacer group (*n* = 64)	Non‐spacer group (*n* = 27)
Age (years)		
Mean (SD)	75.2 (7.8)	74.2 (8.5)
ISUP grade group		
1	5 (7.8)	2 (7.4)
2	26 (40.6)	13 (48.2)
3	14 (21.9)	7 (25.9)
4	5 (7.8)	2 (7.4)
5	13 (20.3)	3 (11.1)
Missing	1 (1.6)	0 (0.0)
PSA (ng/mL)		
Median (Q1, Q3)	7.1 (4.3, 10.5)	6.8 (5.1, 10)
Treatment extent		
Prostate only	0 (0.0)	2 (7.4)
Prostate and seminal vesicles	63 (98.4)	24 (88.9)
Prostate, seminal vesicles and pelvic nodes	1 (1.6)	1 (3.7)
Index lesion location (laterality)		
Right	34 (53.1)	12 (44.4)
Left	25 (39.1)	11 (40.7)
Missing	5 (7.8)	4 (14.8)
Index lesion location		
Apex	18 (28.1)	7 (25.9)
Base	14 (21.9)	5 (18.5)
Midzone	31 (48.4)	11 (40.7)
Missing	1 (1.6)	4 (14.8)

*Note:* Unless otherwise indicated, data are presented as *N* (%).

Abbreviations: PSA, prostate‐specific antigen; Q1, first quartile; Q3, third quartile; RT, radiation therapy; SD, standard deviation.

### 3.2 Spacing and Symmetry

At baseline, a mean mid‐line spacing of 1.04 cm was achieved between the rectum and prostate at the mid‐gland level (Table 2). The mean mid‐line spacing for the apex and base was approximately 0.8 and 0.9 cm, respectively (Table 2).

**TABLE 2:** Midline spacing at baseline and during treatment.

	Apex			Mid‐gland			Base		
	Mean (SD)	MD (95% CI)	*p*	Mean (SD)	MD (95% CI)	*p*	Mean (SD)	MD (95% CI)	*p*
Baseline	0.78 (0.36)	Reference		1.04 (0.28)	Reference		0.85 (0.53)	Reference	
Day 1	0.72 (0.36)	−0.06 (−0.14, 0.01)	0.13	1.06 (0.29)	0.03 (−0.04, 0.09)	0.22	0.97 (0.58)	0.12 (0.01, 0.23)	0.04
Day 5	0.69 (0.37)	−0.08 (−0.16, −0.01)	0.03	1.10 (0.28)	0.06 (0.00, 0.12)	0.05	0.95 (0.62)	0.10 (−0.01, 0.21)	0.06
Day 10	0.75 (0.43)	−0.03 (−0.13, 0.07)	0.57	1.07 (0.29)	0.04 (−0.04, 0.11)	0.13	0.96 (0.61)	0.11 (−0.2, 0.24)	0.12
Day 15	0.71 (0.36)	−0.07 (−0.15, 0.01)	0.09	1.12 (0.26)	0.08 (0.17, 0.15)	0.16	0.94 (0.60)	0.09 (−0.02, 0.21)	0.11
Day 20	0.73 (0.37)	−0.05 (−0.14, 0.04)	0.32	1.10 (0.26)	0.07 (0.00, 0.13)	0.05	0.91 (0.59)	0.06 (−0.05, 0.17)	0.33

Abbreviations: MD, mean difference compared to baseline; SD, standard deviation.

Weekly CBCT analysis demonstrated consistent spacer positioning and anatomical separation throughout treatment. At apex and mid‐gland levels, the measured midline spacing on CBCT during treatment was consistent with the baseline measurement, indicating stability of the spacer position and shape over time. Midline spacing was consistent with baseline at apex, mid‐gland, and base levels during treatment, with no formal evidence of variation across timepoints (joint test: apex Wald *χ* ^2^ = 8.34, *p* = 0.14; mid‐gland Wald *χ* ^2^ = 1.2, *p* = 0.94; base Wald *χ* ^2^ = 6.54, *p* = 0.26). The small absolute mean differences observed at individual timepoints (Table 2) had overlapping 95% CIs and did not represent a consistent temporal pattern.

No significant asymmetry was observed at any level at baseline (Table 3). At the apex level, a small but consistent asymmetry was observed during treatment, with the right side spacing greater than the left side by 0.12 cm, 95% CI 0.06–0.17, with no statistical evidence that the magnitude of asymmetry changed during treatment (Wald *χ* ^2^ = 7.24, *p* = 0.20). At the mid‐gland and base levels, no substantial evidence of asymmetry was observed at any timepoint (Table 3).

**TABLE 3:** Left–right spacing differences at baseline and during treatment.

	Apex			Mid‐gland			Base		
	Mean (SD)	MD (95% CI)	*p*	Mean (SD)	MD (95% CI)	*p*	Mean (SD)	MD (95% CI)	*p*
Baseline	0.00 (0.58)	Reference		0.06 (0.43)	Reference		−0.05 (0.50)	Reference	
Day 1	−0.15 (0.53)	−0.15 (−0.30, −0.01)	0.04	0.08 (0.42)	0.02 (−0.07, 0.11)	0.68	−0.06 (0.47)	−0.01 (−0.15, 0.13)	0.93
Day 5	−0.16 (0.58)	−0.16 (−0.31, −0.01)	0.04	0.07 (0.45)	0.00 (−0.10, 0.12)	0.89	−0.14 (0.47)	−0.09 (−0.25, 0.07)	0.83
Day 10	−0.14 (0.55)	−0.14 (−0.28, −0.01)	0.04	0.05 (0.48)	−0.01 (−0.12, 0.09)	0.72	−0.09 (0.50)	−0.04 (−0.20, 0.13)	0.95
Day 15	−0.09 (0.53)	−0.09 (−0.23, 0.04)	0.17	0.01 (0.49)	−0.04 (−0.16, 0.06)	0.41	−0.06 (0.49)	0.00 (−0.17, 0.16)	0.82
Day 20	−0.14 (0.57)	−0.14 (−0.29, 0.00)	0.06	0.03 (0.47)	−0.02 (−0.14, 0.09)	0.65	−0.06 (0.49)	0.00 (−0.17, 0.16)	0.68

Abbreviations: MD, mean difference compared to baseline; SD, standard deviation.

### 3.3 Dosimetry Outcomes

Differences in PTV and rectal dose distributions between the two cohorts are outlined in Table 4. Rectal dose was consistently lower in the spacer cohort, with statistically significant differences compared to the non‐spacer cohort across all dose‐volume metrics except for the V60Gy, the prescription dose. Additionally, the dose to the PTV (D98%) was significantly higher in the spacer cohort compared to the non‐spacer cohort (57.8 vs. 57.3 Gy, *p* < 0.001).

**TABLE 4:** Dosimetry outcomes.

	Spacer cohort	Non‐spacer cohort	Mean difference (95% CI)	Mean percentage difference (%)^a^	*p*
PTV (mean, SD)					
D98% (Gy)	57.8 (0.5)	57.3 (0.6)	0.5 (0.3, 0.7)	0.87%	< 0.001
Rectum (mean, SD)					
V30Gy (%)	24.2 (11.9)	33.8 (9.6)	−9.6 (−14.8, −4.5)	−28.4%	< 0.001
V40.8Gy (%)	12.3 (8.2)	22.9 (7.4)	−10.6 (−14.2, −6.9)	−46.3%	< 0.001
V50Gy (%)	6.6 (5.4)	15.2 (5.3)	−9.3 (−12.7, −5.9)	−61.2%	< 0.001
V52.8Gy (%)	4.8 (4.4)	12.8 (4.7)	−8.0 (−9.9, −5.9)	−62.5%	< 0.001
V54Gy (%)	4.1 (3.9)	11.6 (4.3)	−7.5 (−9.3, −5.6)	−64.7%	< 0.001
V57Gy (%)	3.4 (7.1)	8.0 (3.3)	−4.6 (−7.5, −1.8)	−57.5%	< 0.001
V60Gy (%)	0.7 (1.4)	1.1 (1.3)	−0.4 (−1.0, 0.2)	−36.4%	0.16
*D*_max_ (point)	60.6 (3.5)	61.7 (0.8)	−1.0 (−1.9, −0.1)	−1.62%	0.03
*D*_max_ (0.035 cc)	59.5 (3.7)	60.7 (0.8)	−1.2 (−0.2, −0.2)	−2.00%	< 0.001

Abbreviations: *D*_max_, maximum dose; DXX%, dose to XX% of structure volume; Gy, gray; PTV, planning target volume; VXXGy, volume receiving minimum of XX Gy.

^a^ Percentage difference was calculated as (mean difference/mean of the non‐spacer cohort) × 100.

### 3.4 Toxicity

No complications related to the spacer insertion procedure were reported and no rectal wall infiltration was observed.

**FIGURE 1:**
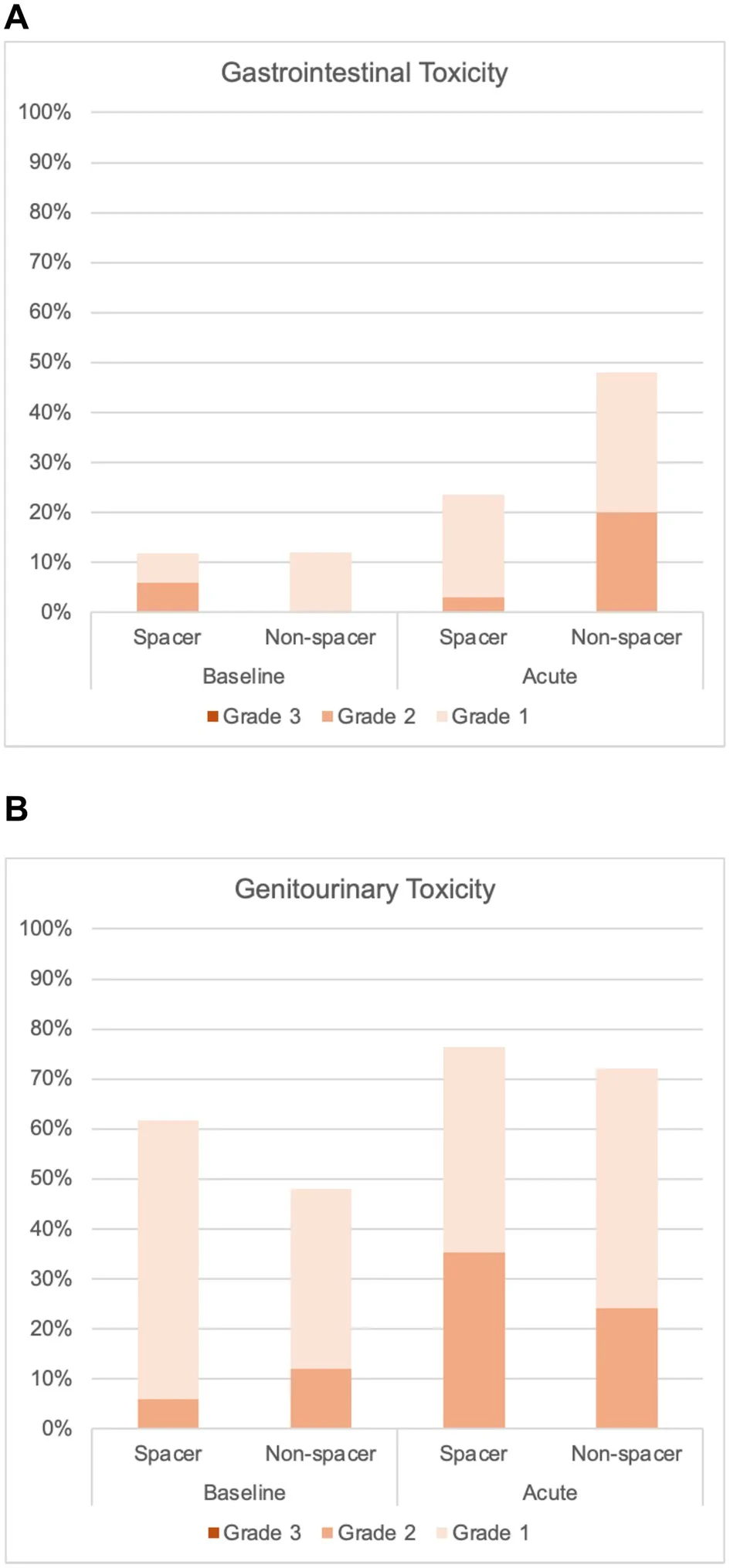
Rates of CTCAE‐defined gastrointestinal toxicity (A) and genitourinary toxicity (B) by grade as recorded at baseline and within 3 months of HFRT (acute).

Thirty‐four and twenty‐five patients in the spacer and non‐spacer cohorts, respectively, had a minimum of 3 months post‐treatment follow‐up data available, and were included in the toxicity analysis. Baseline GI toxicity was comparable between groups (spacer cohort 11.8% vs. non‐spacer cohort 12.0%; odds ratio [OR] = 0.97, 95% CI 0.19–4.81, *p* = 0.98) (Figure 1A). The spacer cohort demonstrated substantially lower rates of acute (< 3 months post HFRT) GI toxicity compared to those without a spacer (23.5% vs. 48.0%; OR = 0.33, 95% CI 0.11–1.01, *p* = 0.05) (Figure 1A), however, this was not statistically significant. Notably, grade ≥ 2 acute GI toxicity occurred in 2.9% of participants who had received a spacer compared to 20.0% in the non‐spacer cohort.

The median (Q1, Q3) toxicity follow‐up for the spacer cohort was 10.0 (7.5, 13.6) months. In the late toxicity period (> 3 months post‐HFRT), the rate of Grade 1 GI toxicity was 14.7%, with no grade ≥ 2 late toxicity reported. By the time of last recorded follow‐up, the rate of grade ≥ 1 toxicity had resolved to baseline levels (11.8%).

Baseline GU toxicity was 61.8% in the spacer cohort versus 48.0% in the no spacer group (OR 1.75, 95% CI 0.61–4.98, *p* = 0.29). No significant differences in acute GU toxicity were observed within 3 months of treatment (76.5% vs. 72.0%, *p* = 0.69).

## 4 Discussion

The findings of this retrospective study contribute to the growing evidence base for the use of sHA‐based spacers to support prostate radiotherapy. In summary, this study demonstrated consistent and stable separation between the rectum and prostate throughout a four‐week course of HFRT, translating to improved rectal dosimetry for patients in the spacer cohort compared to the non‐spacer cohort. Clinically, these dosimetric benefits were associated with a lower incidence of acute GI toxicity, particularly grade ≥ 2 events. Importantly, sHA spacer insertion was well tolerated, with no major complications reported.

This study makes a novel contribution to the literature by providing a systematic assessment of sHA‐based spacer position and symmetry using weekly CBCT imaging throughout the treatment course. Previous studies have predominantly focused on baseline spacing characteristics. Our findings of maintained separation at apex and mid‐gland levels with minimal variation are consistent with the high symmetry and stability rates reported by Svatos et al. [11], demonstrating the stability and reliability of sHA‐based spacers in clinical practice. However, the statistically significant increase in base‐level spacing of 0.1 cm during treatment diverges from the sub‐millimetre stability reported by Svatos et al. [11] at 3 months (0.24 mm at mid‐gland, −0.3 mm at superior plane, 0.59 mm at inferior plane). This discrepancy may be an artefact of the challenging visibility of the spacer at the base level on CBCT compared to MRI.

The apical asymmetry observed in our study during treatment may reflect the recognised technical challenges of achieving adequate apical spacing. Svatos et al. [11] reported a mean apical separation of 9.67 mm (±3.95 mm), highlighting the apex as the most variable region for spacer placement. Nevertheless, the overall stability demonstrated in our weekly CBCT analysis supports the real‐world clinical utility of sHA‐based spacers for maintaining consistent recto‐prostatic separation throughout HFRT and facilitating favourable rectal dosimetry.

The consistent and statistically significant reductions in rectal dose across multiple dose‐volume levels (Table 4) compared to the control cohort in our study reflect the findings of previous studies where a sHA‐based spacer was used [8, 10]. Mariados et al. [8] and Williams et al. [10] report a rectal V53Gy of 1.7% and 2.8%, respectively, compared to a V58.2Gy of 4.8% in our study. The slightly higher rectal dose at a similar dose‐volume level in our study could be due to the rectum being contoured to a lesser superior and inferior extent in our study, as well as differences in planning technique (VMAT vs. IMRT [8]). Further, we observed a small but significant increase in PTV D98% (57.8 vs. 57.3 Gy, *p* < 0.001) in the spacer cohort, which is consistent with prior reports that spacers can facilitate improved PTV coverage [12], however, this difference was not clinically significant.

The observed difference in rectal dosimetry between the cohorts was associated with a significant difference in acute GI toxicity, which is consistent with the literature [8]. The GI toxicity outcomes in this study align with other studies of sHA‐based spacers in the setting of HFRT. The rate of acute grade ≥ 2 toxicity in the spacer cohort (2.9%) was consistent with the 2.9% (4 of 136 patients) reported by Mariados et al. [8]. Late grade ≥ 2 GI was not reported in the spacer cohort of this study, comparing favourably to the study conducted by Chapet et al. [9], which reported late grade ≥ 2 GI toxicity in 12% of patients, though all toxicities in that study had resolved by 36 months. Our study may underestimate the rate of late GI toxicity given the short median follow‐up in the spacer cohort relative to the usual time course of late GI sequalae. Nevertheless, these findings support the reproducibility of spacer benefits in reducing acute GI toxicity during HFRT, with toxicity rates consistently below 5% across multiple studies when sHA‐based spacers are employed. The comparable GU toxicity profiles between spacer and non‐spacer cohorts in our study were expected [8], given that rectal spacers selectively protect posterior structures.

This study has several limitations that should be acknowledged. First, the retrospective design introduces the potential for reporting and documentation bias. Secondly, it is possible that confounding factors may contribute to the observed differences in acute toxicity between the two cohorts. The use of sHA spacers was predominantly at one of the two sites included in this study, which was associated with clinician preference. However, baseline characteristics (Table 1) were comparable between the two cohorts and standardised radiotherapy planning and treatment protocols are used across different sites at our institution. The relatively small sample size, particularly within the control cohort, and wide confidence intervals limit detection of small temporal variations and limit the statistical power of the analysis. Further, the use of only linear measurements may have been insufficient to detect other variations in spacing and symmetry over time, given the apparently small effect sizes. In addition, although acute GI toxicity was well captured, the duration of follow‐up was insufficient to comprehensively assess late effects, which are an important endpoint in prostate radiotherapy. Finally, spacer insertion was performed at a single institution, which may limit the generalisability of these findings to broader clinical practice.

Future research should include pragmatic prospective trials which directly compare sHA‐ to hydrogel‐based spacers to establish comparative efficacy and safety profiles. Given that the utilisation of rectal spacers as part of the standard of care is increasing, this kind of trial might be suited to a registry‐based randomised controlled trial [13] at institutions which use both sHA‐ and hydrogel‐based spacers in routine practice. Further, the incorporation of patient‐reported outcomes in future trials will provide valuable insights into the patient experience and quality of life impacts of sHA‐based spacers in the HFRT setting. Finally, extended follow‐up studies are essential to comprehensively evaluate long‐term GI and GU outcomes, as late toxicities may emerge beyond the current observation periods and influence clinical decision‐making and patient counselling.

In conclusion, sHA‐based rectal spacers have demonstrated safety and efficacy in clinical practice while offering distinct technical advantages. The present data add to the growing body of evidence supporting the routine implementation of sHA‐based spacers in prostate HFRT and contribute novel information regarding the stability and symmetry of sHA‐based spacers over the course of treatment.

## Funding

This study was supported by funding from Palette Life Sciences, now Teleflex (Morrisville, NC, USA), and the Icon Cancer Foundation.

## Ethics Statement

This study was approved by Bellberry Human Research Ethics Committee (approval number: 2023‐10‐1252).

## Consent

The ethics committee granted a waiver of consent for retrospective use of data in this study.

## Conflicts of Interest

The authors declare no conflicts of interest.

## Data Availability

The data that support the findings of this study are available from the corresponding author upon reasonable request.
